# Premature Aging in Chronic Kidney Disease: The Outcome of Persistent Inflammation beyond the Bounds

**DOI:** 10.3390/ijerph18158044

**Published:** 2021-07-29

**Authors:** Andrea Figuer, Guillermo Bodega, Patricia Tato, Gemma Valera, Nadia Serroukh, Noemi Ceprian, Patricia de Sequera, Enrique Morales, Julia Carracedo, Rafael Ramírez, Matilde Alique

**Affiliations:** 1 Departamento de Biología de Sistemas, Universidad de Alcalá, 28871 Alcalá de Henares, Madrid, Spain/Instituto Ramón y Cajal de Investigación Sanitaria (IRYCIS), 28034 Madrid, Spain; andrea.figuer@salud.madrid.org (A.F.); gemmavaar@hotmail.com (G.V.); manuel.ramirez@uah.es (R.R.); 2 Departamento de Biomedicina y Biotecnología, Universidad de Alcalá, 28871 Alcalá de Henares, Madrid, Spain; guillermo.bodega@uah.es; 3 Departamento de Biología de Sistemas, Universidad de Alcalá, 28871 Alcalá de Henares, Madrid, Spain; patricia.tato@edu.uah.es; 4 Departamento de Genética, Fisiología y Microbiología, Facultad de Ciencias Biológicas, Universidad Complutense de Madrid/Instituto de Investigación Sanitaria Hospital 12 de Octubre (imas12), 28040 Madrid, Spain; nadiaseroukh@gmail.com (N.S.); nceprian@gmail.com (N.C.); 5 Sección de Nefrología, Hospital Universitario Infanta Leonor, 28031 Madrid, Spain; patricia.desequera@salud.madrid.org; 6 Departamento de Medicina, Universidad Complutense de Madrid, 28040 Madrid, Spain; emoralesr@senefro.org; 7 Departamento de Nefrología del Hospital Universitario 12 de Octubre, Instituto de investigación i+12, 28041 Madrid, Spain

**Keywords:** aging, cellular senescence, chronic kidney disease, elderly, frailty, oxidative stress

## Abstract

Over the last hundred years, life expectancy in developed countries has increased because of healthier living habits and the treatment of chronic pathologies causing premature aging. Aging is an inexorable, time-dependent, multifactorial process characterized by a series of progressive and irreversible physiological changes associated with loss of functional, psychological, and social capabilities. Numerous factors, such as oxidative stress, inflammation, and cellular senescence, and an irreversible geriatric syndrome known as frailty, contribute to human body deterioration in aging. The speed of aging may differ between individuals depending on the presence or absence of multiple factors (genetic and/or environment) and the subsequent misbalance of homeostasis, together with the increase of frailty, which also plays a key role in developing chronic diseases. In addition, pathological circumstances have been reported to precipitate or accelerate the aging process. This review investigated the mechanisms involved in the developing pathologies, particularly chronic kidney disease, associated with aging.

## 1. Introduction

### Aging: A Physiological Stage Associated with a Higher Frequency of Pathologies

The aging process has well-defined characteristics in all living beings. Aging and the progressive physiological changes in an organism lead to senescence, a decline of biological function, and the organism’s ability to adapt to metabolic stress. Physiological aging is accompanied by an alteration of numerous biochemical parameters and a progressive decline affecting different organ systems, significantly affecting individual and social behavior [1]. All these changes worsen a person’s health status and reduce their quality of life.

The term “life expectancy” refers to the number of years a person can expect to live. Over time, the average human life expectancy has increased about 3 months per year, resulting in approximately 25 years to approximately 65 years in men and 70 years in women. This mortality reduction is mainly due to improvements in health, nutrition, education, income, health status, and medicine. Higher population longevity results in higher rates of aged people because age, as a chronological measure, is an important risk factor in multiple diseases, known as age-related diseases, including cardiovascular diseases, diabetes, and kidney diseases [2,3,4]. In the last 160 years, we have witnessed an improvement in the global life expectancy, which represents the delaying of the mean age of death. Despite life expectancy and aging being related, they have different means, and it is known that an increase in life expectancy can increase with age [5]. The World Health Organization (WHO) stated that age-related diseases increased in the last century due to the increase in lifespan, expecting a doubling of the world’s population aged over 60 years by the year 2050.

There are two types of aging—natural aging (biological or physiological) and premature aging (pathological). Natural aging occurs when a person suffers inevitable age- and time-associated changes [6,7]. The physiological aging process is very heterogeneous, even among individuals of the same species. Differences in the aging rate have led to the definition of “biological age,” which is associated with the functional status of organ systems, regardless of the person’s chronological age [8,9]. The factors that influence the physiological aging process can be classified as either intrinsic (such as inheritance, sex, and race) or extrinsic (such as environment, toxic habits, diet, and physical activity) [10,11,12]. Longitudinal studies have been conducted in healthy persons of different “omics,” and different aging patterns (ageotypes) according to the changes in the molecular routes that have been described [13,14].

In contrast, pathological aging is marked by various physical and mental disorders, usually associated with physiological aging at earlier ages. These alterations are attributed to acute and/or chronic diseases, environmental factors, toxic habits (such as smoking and consumption of alcohol and other drugs), and cancer [15,16,17].

Physiological aging is a consequence of the functional decline of cells, tissues, and organs, causing characteristic changes throughout the life cycle. Aging is determined by genetic and socio-physiological or environmental factors such as diet, mental stress, education, socioeconomic status, sedentary lifestyle, and/or substance abuse [5]. Both can be modulated in part, especially the environmental component that directly or indirectly regulates the genetic factor playing a key role in the aging process. It has been described that physical exercise is fundamental to counter premature aging. After all, it can activate mechanisms that prevent aging [18]. As previously mentioned, the physiological aging process and longevity of the species are genetically regulated; however, human longevity is only inheritable by about 15–40%, with a recent estimate of 16% [19]; thus, environmental factors (84%) are superior to genetic burden (16%) as decisive factors in aging.

**Figure 1:**
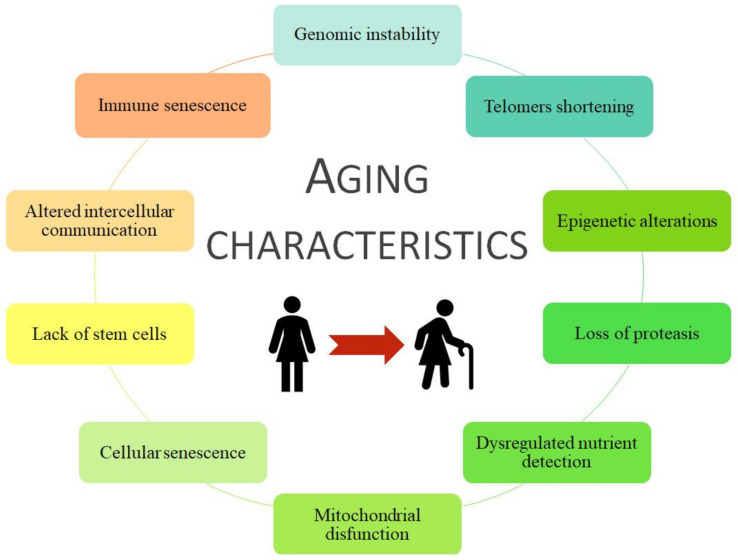
The 10 characteristics of aging. Modified from [27].

Aging is a haphazard process lead by multiple factors, socio/physiological/lifestyle/environmental circumstances, that humans are kept in continuous contact with, and therefore they could change our aging process during the development and the reproductive years. Aging is associated with more frequent development of chronic disease pathologies, such as cancer, infectious diseases, diabetes mellitus, obesity, metabolic syndrome, osteoporosis, neurodegenerative diseases, chronic kidney disease (CKD), and cardiovascular disease (CVD) [20,21,22]. Moreover, some age risk factors are smoking, hyperglycemia, hyperlipidemia, obesity, hypertension, dyslipidemia, and loss of bone density and frailty, grouped as metabolic risk factors that can lead to premature death [23,24,25]. All of them are considered metabolic risk factors that join with physical inactivity cause premature aging and the development of associated pathologies such as CVD and/or CKD. Coronavirus disease (COVID-19) is an infectious disease associated with worse effects and higher mortality in older people or those with comorbidities, i.e., those with a higher frailty rate [26]. In general, specific damages to organ systems during aging are characterized by 10 common traits (Figure 1).

Changes with older age cause deterioration in the kidney and other vital organs. Among the many pathologies associated with aging, CKD and CVD are some of the most common ones, with the latter being the main cause of global morbidity and mortality [28,29].

## 2. Frailty and Pathologies Associated with Aging

The accumulation of health problems increases vulnerability, which is different for each individual [30]. To define vulnerability, the term frailty was coined [31], as frail individuals exhibit a higher mortality rate than healthy individuals of the same age.

Frailty is one of the most well-known geriatric syndromes, which is more prevalent in females than males (with frailty percentages of 48.8% vs. 41.8%) [32,33] and is related to age and lower ability to maintain homeostasis. It is characterized by weakness, sarcopenia, reduced stress response, and low physical activity and resistance [34]. The higher frailty prevalent susceptibility in women versus men reduces physiological function and vests a greater risk of developing age-related diseases such as CVD associated-CKD [35].

There are different methods of assessing frailty in a person (Table 1). However, the Frailty Phenotype and Frailty Index of Accumulative Deficits are the most accepted and validated tests [36]. In general, the frailty rate increases with age, reaching the peak of 85 years or more [30,37,38].

Age-related comorbidities increase the frailty rate. This phenomenon occurs in CKD, correlating with the appearance of comorbidities such as diabetes mellitus and CVD [26,39,40].

**Table 1:** Frailty measurement test.

Test	Description	Reference
**Frailty phenotype (Fried criteria)/Cardiovascular health study**	Unintentional weight loss >4.5 kg in the last year Weakness (low grip strength) Fatigue and low resistance Slowness Low physical capacity	[41]
**Frailty index of accumulative deficits**	≥30 symptoms, disease, disabilities, comorbidities, or health deficiencies Expressed as a ratio (for example 3/30 = 0.1)	[37]
**Vulnerable elders survey**	13 questions about age, self-perceptions of health, needing assistance in daily activities, and physical ability A patient with a score of ≥3 is considered vulnerable	[42]
**Sarcopenia (loss of muscle due to aging)**	Rectus femoris cross-sectional area by ultrasound Computed tomography of the left and right psoas muscles at the L4 vertebra	[43,44]
**Frailty index derived from the comprehensive geriatric assessment**	Clinical analysis of medical, nutritional, functional, and psychological variables Initially 10 domains, but later expanded to 52 domains	[45,46]
**Edmonton frailty scale**	Evaluation of 17 variables on cognition, general health status, self-reported health, functional independence, social support, polypharmacy, mood, continence, and functional performance A person with a score >5 is considered frail, with different severity depending on the score: vulnerable (6–7), mildly frail (8–9), moderately frail (10–11), and severely frail (12–17)	[47]
**Tilburg frailty indicator**	15 self-reported items evaluating: physical components (weight loss, balance, difficulty in walking, health, gripping, vision, and tiredness), psychological factors (memory, anxiety, coping mechanisms and feeling down), social elements (living conditions, social isolation, social support) A person with a score of ≥5 is considered frail	[48]

## 3. Chronic Kidney Disease

### 3.1. Brief Description of Chronic Kidney Disease

CKD is a progressive and irreversible condition whose definitions and classification have evolved over time. The international guidelines currently define CKD as a glomerular filtration rate (GFR) of <60 mL/min/1.73 m^2^ or evident structural kidney damage observed using diagnostic techniques for >3 months [49].

Diabetes and hypertension are the main etiologies of CKD worldwide [50]. However, regardless of the underlying cause, the functional and structural alterations associated with renal aging are considered a primary pathogenic mechanism in CKD development. Thus, CKD has become the main warning sign of premature aging, associated with high morbidity and mortality rates and high economic costs due to both drug treatment and renal replacement therapy [51,52]. CKD currently affects 10% of the adult population [49,50], but this percentage will be increased with the predicted population aging, with a devastating impact on patients and family members.

### 3.2. Chronic Kidney Disease during the Process of Physiological Aging

After the fourth decade of life, there is approximately 10% loss of renal parenchyma with each succeeding decade (from an overall weight of >400 g during the third to fourth decades of life to an overall weight of <300 g in the ninth decade of life), as the reduced proliferation of renal epithelial cells leads to renal cortical thinning. In addition, the number of functional nephrons decreases, causing glomerular basement membrane thickening along with hyaline deposits, capillary collapse, and tubulointerstitial fibrosis. By the sixth decade of life, renal tubular function decreases by approximately 20%, causing tubular atrophy [53].

Structural changes induce functional changes. Over time, the GFR decreases to about 50 mL/min and creatine clearance <65 mL/min, respectively [54,55]. It has become a necessity to actualize the definition of CKD, including age-specific GFR thresholds. Fewer healthy elderly diagnosed with CKS could contribute to reducing inadequate care and its associated adverse effects [56]. Renal aging is also characterized by decreased tubular function, resulting in reduced urine concentration capacity [54] and sodium reabsorption capacity in the ascending loop of Henle. In addition, there is deterioration in renal medullary tonicity, reducing the free water reabsorption capacity of the collecting tubules in the antidiuretic state [54].

The renin–angiotensin–aldosterone axis, a key factor in sodium and potassium regulation, and blood pressure are also altered. The juxtamedullary apparatus of the kidney secretes renin. In elderly individuals, plasma renin and aldosterone levels are reduced by 40–60% and 30–50%, respectively [57,58], thereby decreasing potassium excretion capacity and increasing the risk of hyperkalemia and sensitivity toward drugs that inhibit urinary potassium excretion, such as potassium-sparing diuretics [59].

The structure and function of the renal vasculature undergo the following alterations: arterial wall thickening and glomerulosclerosis due to extracellular matrix deposits and occlusion of the afferent and efferent arterioles, reducing the number of functional glomeruli [53]. In addition, there is an increase in sympathetic tone, increasing vasoconstriction at the glomerular level. As a result, renal vasodilator substances, such as atrial natriuretic peptide and nitric oxide (NO), lose their effectiveness. NO production is decreased, affecting maintenance of renal plasma flow, particularly in older men [54].

Klotho, a transmembrane protein acting as a cofactor of fibroblast growth factor 23 [54], regulates the mechanisms involved in systemic renal aging. Klotho is involved in the metabolism of both calcium/phosphorus and vitamin D, is produced by the proximal tubule cells, and is released into the blood, where it acts as a hormone regulating mitochondrial oxidative stress and reducing kidney damage [53]. Klotho also helps protect vasculature since studies in mice have shown that Klotho deficiency contributes to the appearance of vascular calcification [60]. Albuminuria, present in patients with CKD, decreases Klotho expression [61]. During the aging process, there is a decrease in the expression of Klotho in the kidney [62]. This decrease in expression may be due to the inflammatory state as pro-inflammatory cytokines such as tumor necrosis factor-α (TNF-α) and TWEAK reduce Klotho expression through a mechanism dependent on nuclear factor kappa-light-chain-enhancer of activated B cells (NF-κB) [63]. In previous studies, decreased Klotho expression in mice led to various systemic phenotypes resembling human aging.

**Figure 2:**
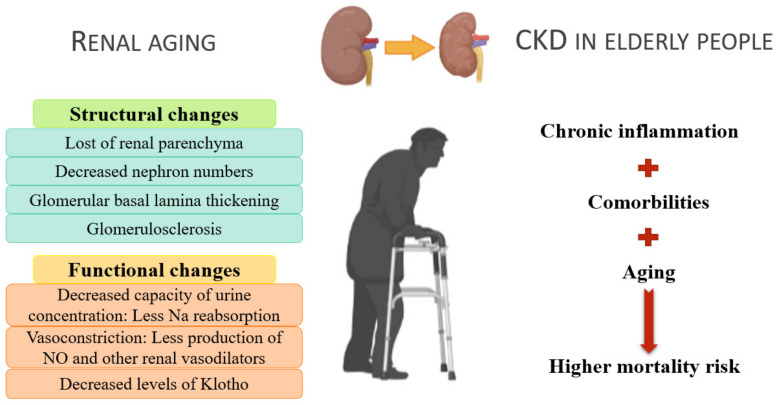
Renal aging and the consequences of chronic kidney disease in elderly individuals.

All these processes are summarized in Figure 2.

## 4. Premature Aging in Chronic Kidney Disease: A Process Associated with an Increase in Cardiovascular Pathologies

The characteristics of CKD are similar to those of the aging process; therefore, it has been hypothesized that CKD promotes premature aging associated with related diseases [64]. Furthermore, chronic diseases usually observed in aging, such as CVD, inflammation, vascular calcification, mineral, and bone disorders, and chronodisruption (chronic alteration of circadian rhythms), are markedly frequent in patients with CKD [25].

CVD is the most clinically relevant comorbidity associated with CKD [65]. The coexistence of both diseases could be explained by the following: (1) patients with CKD have a higher prevalence of non-traditional cardiovascular risk factors, (2) many cardiovascular risk factors exacerbate CKD progression, and (3) CKD itself can be considered a risk factor for CVD [66]. According to 2013 data from the U.S. Renal Data System, an estimated 43% and 15% of patients with CKD experience heart failure and acute myocardial infarction in their lifetime (versus healthy persons: 18.5% and 6.4%, respectively). In addition, CVD is the most important cause of mortality in patients with CKD undergoing dialysis. Mortality from cardiovascular problems is estimated to be two times higher in CKD stage 3 patients and three times higher in stage 4 patients than healthy subjects [66]. Cardiovascular mortality is inversely proportional to the GFR. Also, a higher CVD incidence has been observed in patients undergoing hemodialysis replacement therapy than in those undergoing peritoneal dialysis [66]. Moreover, the accumulation of uremic toxins from the renal pathology leads to chronic inflammation and an increase in oxidative stress, contributing to CVD development as damaged endothelial cells eventually become senescent [29,67].

The development of CVD in patients with CKD is due primarily to endothelial dysfunction [66]. Endothelial cells in patients with renal disorders experience premature senescence due to received stress signals, which may lead to apoptosis [68]. Under physiological conditions, endothelial cells have a non-adherent and anticoagulant surface; however, molecules expressed on the surface of damaged endothelial cells may be altered, increasing cell adhesion capacity [69]. Platelets bind to the damaged surface, triggering the onset of coagulation with consequent inflammation and thrombosis, thereby causing cardiovascular accidents [35].

Several factors, such as inflammation, oxidative stress, primary diseases such as hypertension or diabetes, and hyperlipidemia, contribute to endothelial deterioration in CKD. Another example is hyperphosphatemia, which is present in many patients with renal disorders. High phosphate concentrations increase oxidative stress and reduce the concentration of NO [70,71,72], which the endothelial cells release to relax and avoid the rigidity of the arteries and regulate endothelial permeability. In patients with CKD, NO synthesis decreases because the enzyme endothelial nitric oxide synthase (eNOS) expression is inhibited. Protein kinase C activation increases reactive oxygen species (ROS) production and inhibits eNOS expression [73].

Different potential therapeutic targets have been studied that are altered in uremic patients, highlighting the endocrine phosphate-fibroblast growth factor-23-klotho axis, the nuclear factor erythroid 2-related factor 2 (which regulates mitochondrial function and oxidative stress production), and molecules implicated in the mitochondrial biogenesis and the onset of cellular senescence [74].

## 5. Traditional Factors Involved in Accelerated Aging Induced by Chronic Kidney Disease

### 5.1. Oxidative Stress

Oxidative stress is defined as the accumulation of highly oxidizing molecules, including ROS, either by promoting or inhibiting the body’s antioxidant mechanisms. ROS have partially reduced oxygen metabolites with a high oxidizing capacity, and thus, they can oxidize different molecules, damaging them [75,76]. ROS are generated mainly in the mitochondrial respiratory chain by enzymes such as NADH oxidase (NOX), whose isoforms NOX1, NOX2, and NOX4 have been related to oxidative stress, worsening vascular function, and fibrosis [77,78].

Patients with uremia have an altered balance between prooxidant and antioxidant factors. In addition, the correlation between increased oxidative stress and disease progression has been observed in the early stages of CKD [79,80].

ROS contribute to inflammation during impaired renal function [69,81] as they alter cellular structures and metabolic pathways. Advanced glycation end products (AGEs) are a biomarker for measuring oxidative stress. When attached to the receptor system for AGEs, they generate signaling via MAP kinases, the nuclear internalization of NF-κB subunit p65, leading to increased release of cytokines and pro-inflammatory enzymes and increased expression of adhesion molecules like VCAM-1 [82,83]. At the same time, activated pro-inflammatory immune cells release oxidizing components, thus creating a vicious cycle amplifying oxidative damage [84].

To mitigate the oxidative stress in these patients, antioxidant substances are increased through aerobic exercise and diet (increased vitamin C/E/K and selenium consumption). However, these patients have dietary restrictions, making it difficult for them to consume these nutrients properly. In addition, vitamin C and selenium are lost during dialysis treatment [82,85,86]. On the one hand, vitamin K can mitigate oxidative stress and prevent DNA damage, senescence, inflammation, and aging, which explains why vitamin K deficiency is associated with higher mortality risk in patients with CKD in stage 5 [86,87]. On the other hand, it may seem controversial to recommend physical activity to avoid oxidative stress. It is true that exercise increments ROS production, inflammation, and fatigue however, this reaction is beneficial in the long term because it has been demonstrated that it improves antioxidant defenses and lowers lipid peroxidation levels in the young and the elderly [88].

The relationship between chronic inflammation and aging has led to the coinage of the term “inflamm-aging,” which can also be correlated with frailty syndrome [89]. The involvement of free radicals and mitochondrial stress in the aging process has long been proven and to be widely recognized [90]. This theory, grouped with immunosenescence, forms the theory of “oxi-inflamm-aging” [91]. In chronic oxidative stress, the most affected systems are those in charge of maintaining body homeostasis—the nervous, endocrine, and immune systems. Therefore, disturbances, such as oxidative stress, trigger a response in functioning hemodynamic systems to eliminate the threat. However, damaged homeostatic systems due to CKD do not reduce the same disturbance, but they exacerbate it [13,91]. In this case, inflammation leads to immunosenescence, which affects the other homeostatic systems by further increasing inflammation and oxidative stress, leading to frailty and loss of adaptability, thereby putting the person’s health at risk [92,93].

In addition, decreased renal function can also increase inflammation [92]. In patients with CKD, there is the retention of AGEs [13] and protein oxidation [94], and pro-oxidant molecules [80], which contribute to creating the pro-inflammatory environment.

### 5.2. Inflammation

Decreased renal function in patients with CKD results in the accumulation of uremic toxins in the bloodstream. In addition, other factors such as renal replacement treatments or recurrent infections induce low-level, subclinical chronic inflammation without signs of acute inflammation [95,96,97,98]. This persistent inflammation may cause early aging and higher mortality in patients with CKD than in individuals without CKD of the same chronological age.

Inflammation is maintained by moderate levels of pro-inflammatory mediators such as C-reactive protein (CRP) and cytokines [99] such as interleukin (IL)-6, IL-1, and TNF-α [87,100,101]. As a biomarker of mortality, CRP level is superior to ferritin level and total white blood cell count and comparable to hypoalbuminemia [102]. Furthermore, the increase in CRP is correlated with higher mortality in patients undergoing hemodialysis.

IL-6, IL-1, and TNF-α are directly related to CKD severity. IL-6 is a predictive biomarker of atherosclerosis as it contributes to the generation of atherosclerosis through metabolic, pro-coagulant, and endothelial mechanisms [103]. In addition, IL-1 and IL-6 cause parathyroid hormone inhibition, which is associated with malnutrition, inflammation, cachexia, low bone turnover, and increased mortality in patients undergoing hemodialysis [104,105]. These characteristics are also observed in people with frailty. In particular, IL-6 is associated with depression in patients with advanced CKD, reducing nutritional intake [106].

Moreover, indoxyl sulphate (IS), a uremic toxin found in larger amounts in patients with CKD, binds to the aryl hydrocarbon receptor of monocytes inducing TNF-α secretion, which in turn induces CX3CL1 expression in endothelial cells with the CXCR1 ligand and CD4+/CD28− T cells (most abundant in patients with renal disease). Through T-cell receptor signaling, these T-lymphocytes can cause endothelial cell apoptosis and accelerate CVD progression [107], promoting an inflammatory state and consecutively activating the endothelium and boosting vascular damage. Precisely, inflammation activates Th1 lymphocytes, which release metalloproteases that weaken the fibrous capsule of atheroma plaques, which are generally stabilized through fibrosis and calcification [108], resulting in the formation of unstable plaques whose rupture triggers its release into the blood and causes arterial thrombosis. This increases the risk of CVD in patients with uremia [109]. Furthermore, TNF-α also triggers the expression of the receptor activator of NF-κB ligand, an osteoclast activator, increasing the risk of bone fracture, which is commonly seen in patients undergoing hemodialysis [110]. In addition, TNF-α is associated with malnutrition, inflammation, and mortality [111], which are factors used to describe frailty.

The presence of inflammatory markers is a consequence of not only renal pathology but also renal replacement techniques. During dialysis, an inflammatory response occurs at the systemic level due to the production of pro-inflammatory and anti-inflammatory cytokines. The continuous irritation of the peritoneum during peritoneal dialysis activates genes related to adaptive immunity, promoting the response of Th2 lymphocytes [112]. In hemodialysis, the use of non-biocompatible membranes or non-sterile dialysis fluids contributes to inflammation [95], activating monocytes, and releasing pro-inflammatory cytokines [69]. Depending on the dialysate used, there may be an increase in oxidative stress related to increased specific pro-inflammatory cytokines and NO synthesis [113]. Furthermore, in vitro studies have shown that elevated magnesium concentrations in the acetate dialysis fluid increase ROS production and lipid peroxidation. However, this serves as protection against oxidative stress when other dialysates are used (such as citrate or a mixture of citrate and acetate) [114].

As previously mentioned, the numerous mechanisms that cause inflammation in patients with CKD are not fully known. Therefore, inflammation may also be considered a cause, not only a consequence, of CKD.

## 6. Novel Factors Implied in Accelerated Aging Induced by Chronic Kidney Disease

### 6.1. Epigenetic Factors: Extracellular Vesicles and microRNAs

Extracellular vesicles (EVs) constitute the local and systemic cell-to-cell communication systems [115]. They are very diverse and can be found in all body fluids, and they intervene in physiological and pathophysiological processes as intercellular mediators [116].

The mechanisms underlying the increase in endothelial-derived EV levels increase in patients with CVD or endothelial dysfunction are related to endothelial cell activation in response to factors that alter vascular endothelial stability, such as uremia or inflammation [117,118].

EVs carry proteins, lipids, and nucleic acids, such as microRNAs (miRNAs), which can also be found freely in the blood [119]. These miRNAs are small-sized RNAs (approximately 19–25 nucleotides), and their primary function is to regulate protein expression by binding to messenger RNAs, inhibiting its translation, or facilitating its degradation [120]. Overexpression of miRNA in patients with uremia suggests an immune disorder as the reduced miRNA-155 expression in post-HD patients is considered a good predictor of treatment efficacy [121]. Further, miRNA-155 expression is related to inflammation, particularly IL-6 expression, in patients with CKD [121] and is important for immune response, inflammation, and hematopoietic cell formation due to its ability to shape transcriptase on activated myeloid and lymphoid cells [122]. Therefore, overexpression of miRNA in patients with uremia suggests an immune disorder as the educed miRNA-155 expression in post-HD patients is considered a good predictor of treatment efficacy [123].

Endothelial cells cultured in the presence of IS release more EVs, modulate the expression of miRNAs, and acquire a senescent phenotype. When other endothelial cells are cultured in a medium containing EVs in the presence of a uremic toxin, they inactivate IκB and activate the pro-inflammatory transcription factor NF-κB. The molecules responsible for regulating the expression of IκB may be miRNAs present in EVs. For example, miR-4454, miR-181a-5p, and miR-126-3p can regulate the expression of p53 and NF-κB. Specifically, NF-κB promotes the expression of p53, a well-known tumor suppressor that plays a crucial role in the apoptosis process [117].

Further studies have shown that EVs also promote vascular calcification by affecting vascular smooth muscle cells through modulation of the expression of pro-inflammatory genes and the genes involved in the calcification process [124]. This may also be the case with other uremic toxins, such as p-cresyl, which causes continuous endothelial damage, in which, therefore, the cells respond by increasing the release of EVs [125]. Therefore, endothelium-derived EVs are considered to have a pro-inflammatory and pro-coagulant role [118].

### 6.2. Genomic Damage and Cellular Senescence Induced by Chronic Kidney Disease

Senescent cells acquire a senescence-associated secretory phenotype (SASP), enabling them to secrete a set of chemokines, pro-inflammatory cytokines, and proteases, which activate immune cells (mainly macrophages and monocytes) eliminating the same senescent cells. In older individuals, senescent cells accumulate, partly due to immunosenescence, triggering an inflammatory state [126,127].

In CKD patients, the stress-induced premature senescence phenotype appears because of damage and stress-like uremic toxins, while the SASP phenotype appears due to natural aging [128,129]. In vivo studies realized in renal tubular endothelial cells show that the complement also plays a key role in premature aging, concretely in the acquisition of a senescence phenotype by the cells. The complement can be activated in cases of acute renal injury, releasing C5a. This molecule can modify the DNA methylation of the renal tubular endothelial cells, affecting the expression of proteins implicated in the aryl hydrocarbon receptor signaling, cell cycle regulators, and inducing the expression of molecules implied in the Wnt/β-catenin pathway. The result in the cells is the acquisition of a premature senescence phenotype denoted by the β-galactosidase positivity and the upregulation of the cytokines implicated in the SASP [130].

Patients with CKD undergo a process of accelerated aging and cellular senescence that occurs partly in response to DNA damage by the action of accumulated uremic toxins [126]. During CKD, the kidney loses the ability to remove toxins, such as β-2 microglobulin protein, indoles (3-indoxyl sulphate), phenols (p-cresol sulphate), and guanidines (guanidinosuccinic acid), which build up in the blood [131]. In addition, some toxins (such as hydroquinone, hydroxyl sulphate, methylglyoxal, and leptin) have mutagenic or genotoxic effects. Similarly, other non-uremic toxins, such as trihalomethanes, accumulate and are not eliminated in patients with CKD [131].

The other molecules that cause genomic damage are free radicals and AGEs, especially in diabetic nephropathy. The AGEs are produced during non-enzymatic glycation (Maillard reaction) between reducing sugars such as glucose and proteins, nucleic acids, or lipids [82]. In patients with CKD, genomic damage occurs in the early stages of the disease owing to the presence of some biomarkers, such as 8-hydroxy-2-deoxyguanosine in leukocytes and increased micronuclei, in addition to the presence of chromosomal abnormalities and alterations in mitochondrial DNA [82].

**Figure 3:**
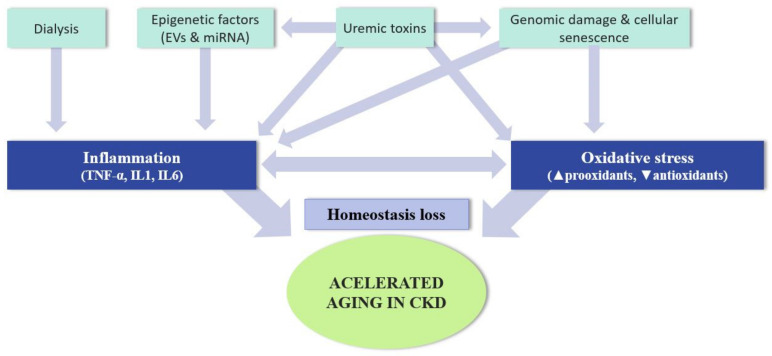
Causes of accelerated aging in patients with chronic kidney disease.

Figure 3 lists the most important factors involved in inflammation and its maintenance in CKD.

## 7. Conclusions

Aging has become the most important risk factor in the main human pathologies, including cancer, diabetes, CVD, neurological disorders, and other chronic diseases, such as CKD. The increase in life expectancy has led to a higher prevalence of these diseases. Given their enormous socioeconomic and medical burden, the search for treatment strategies for these pathologies that accelerate the aging process, such as CKD, has become a preferred field of research. Hence, all strategies to achieve healthy aging can also be of interest in patients with chronic pathologies. Therefore, it is necessary to intensify knowledge on all physiological or pathological aging factors as they may be of enormous interest in both fields.
